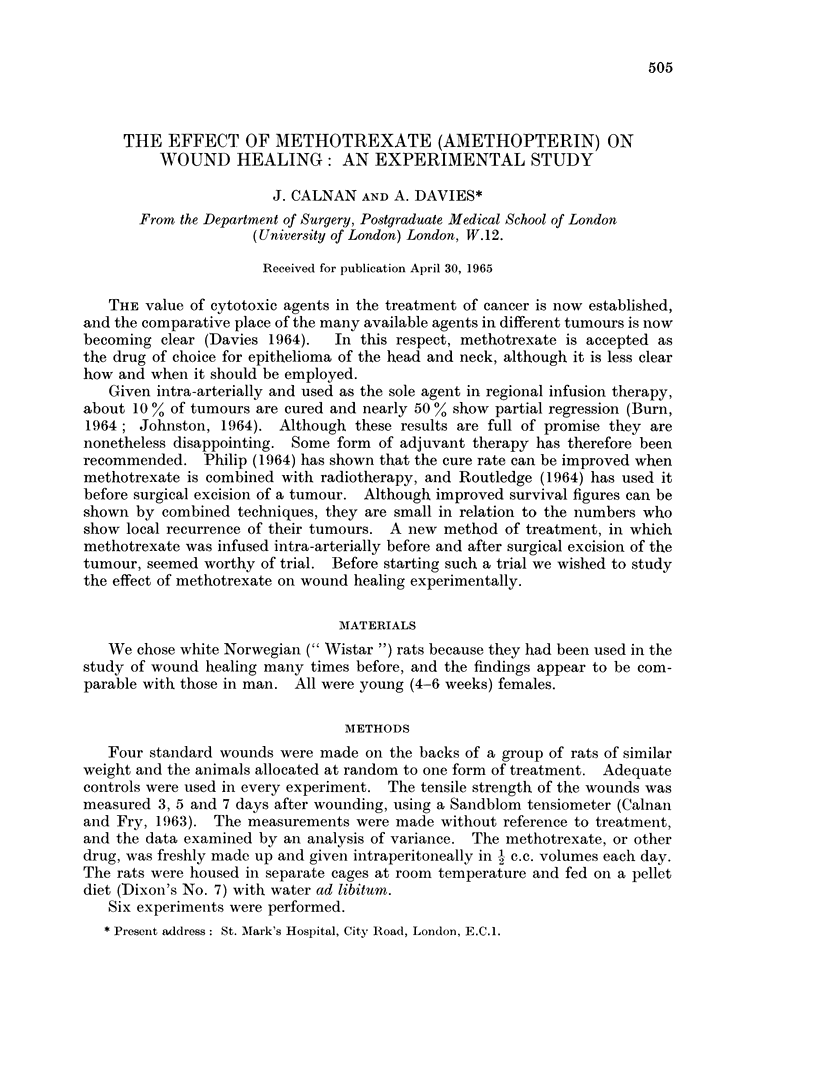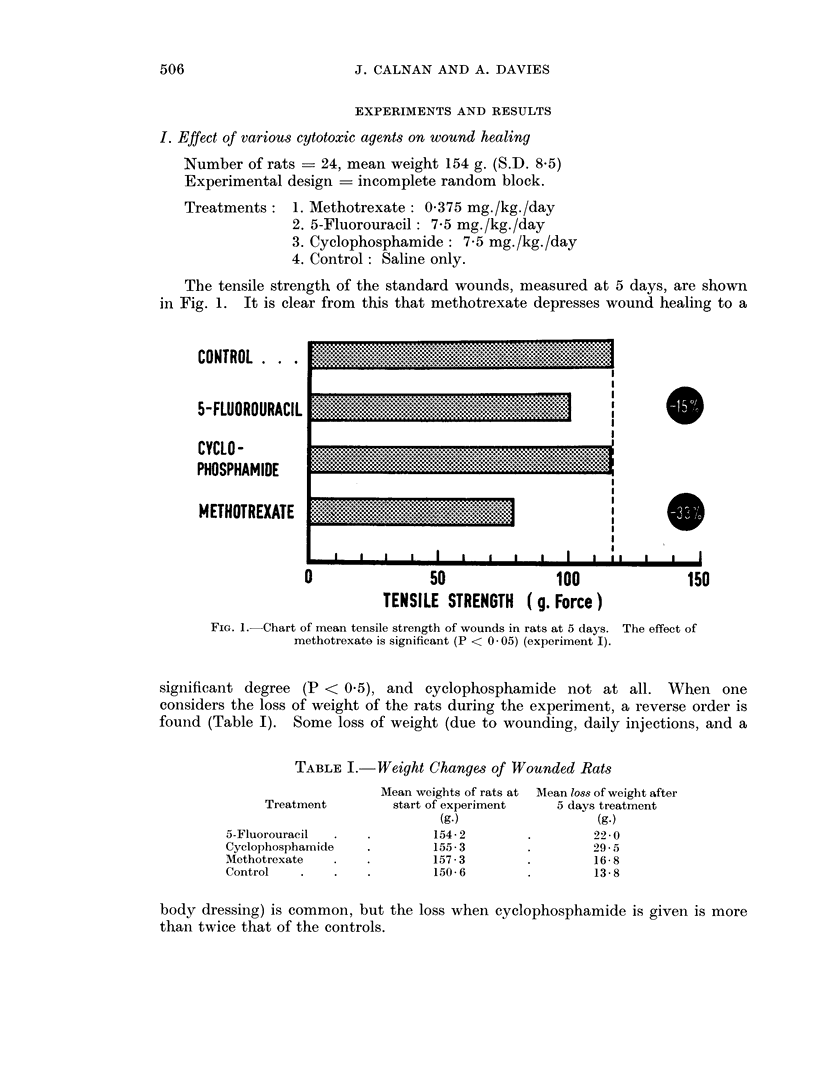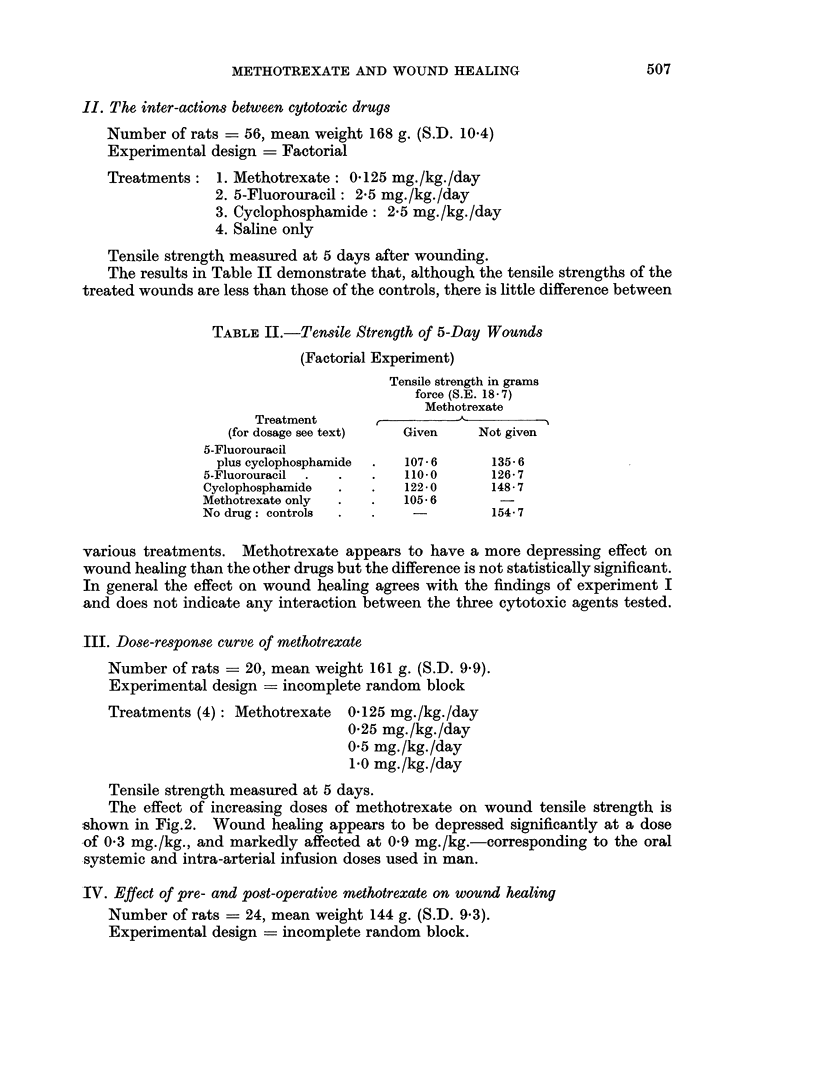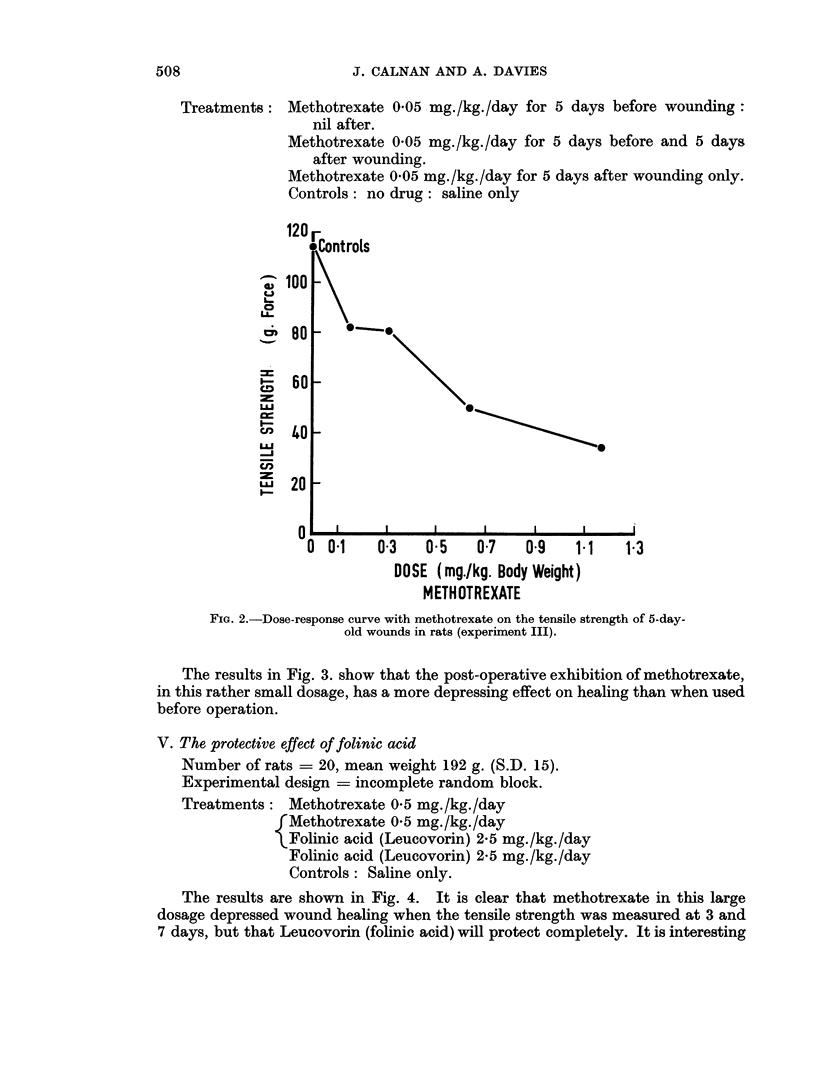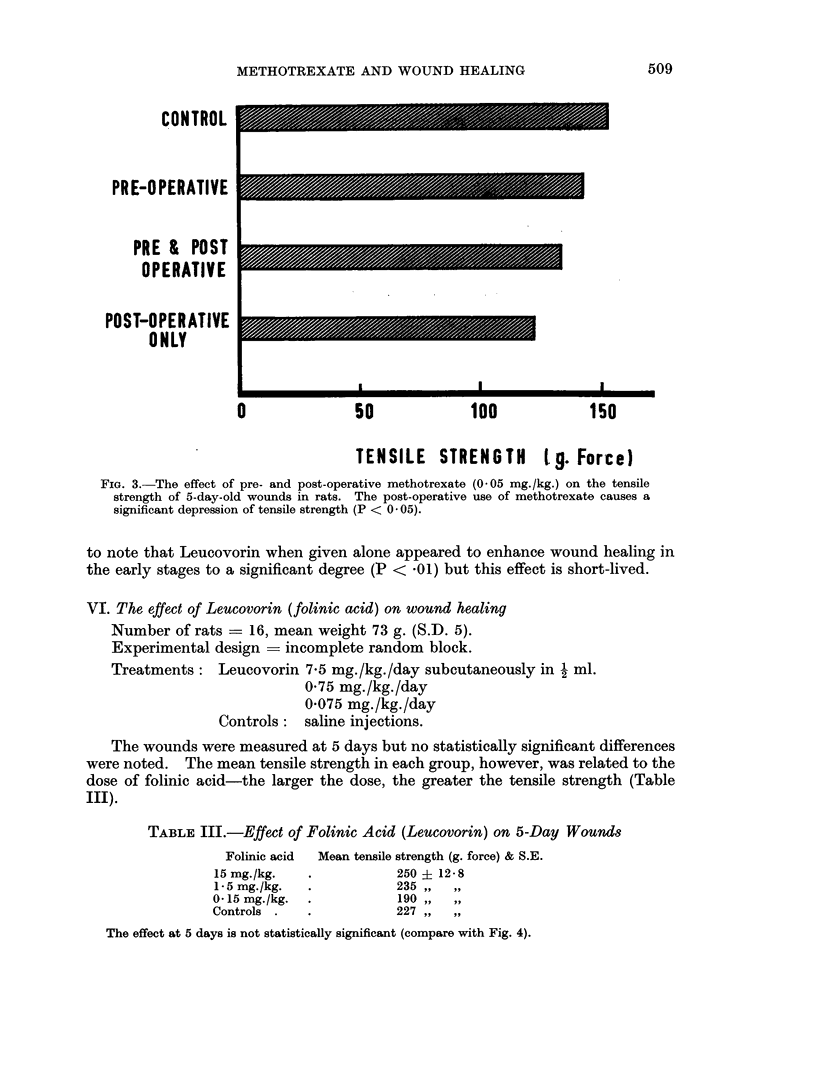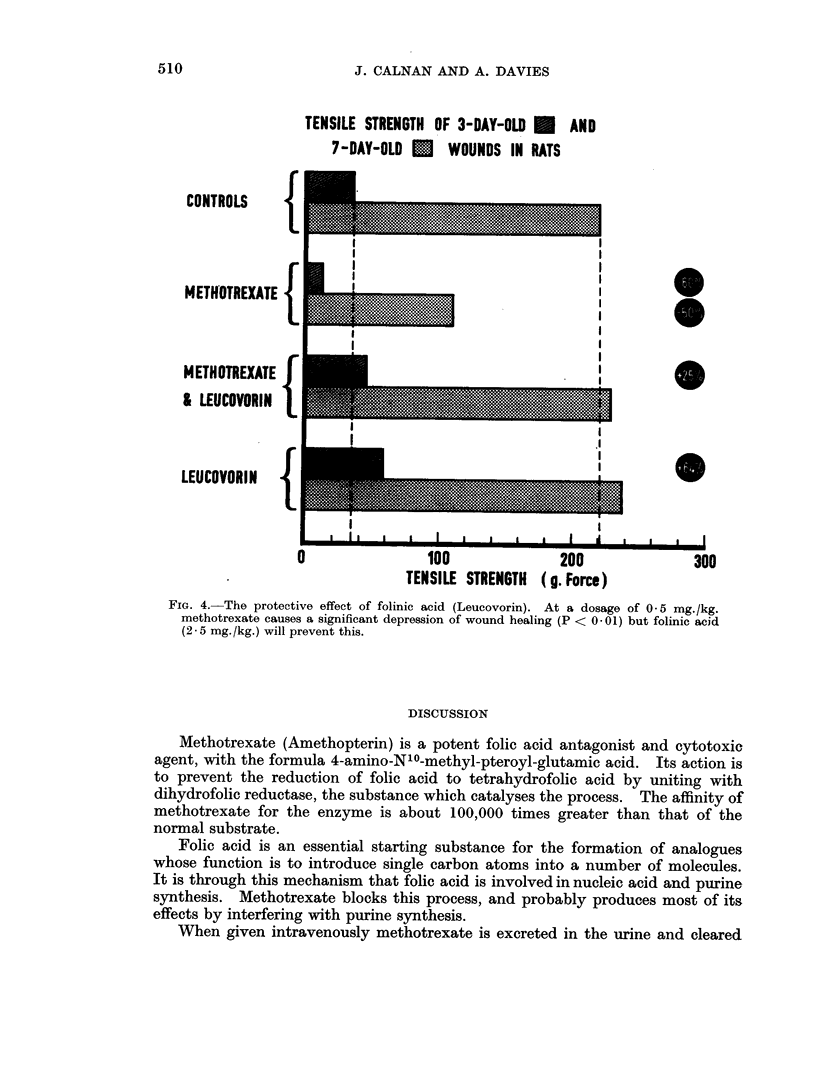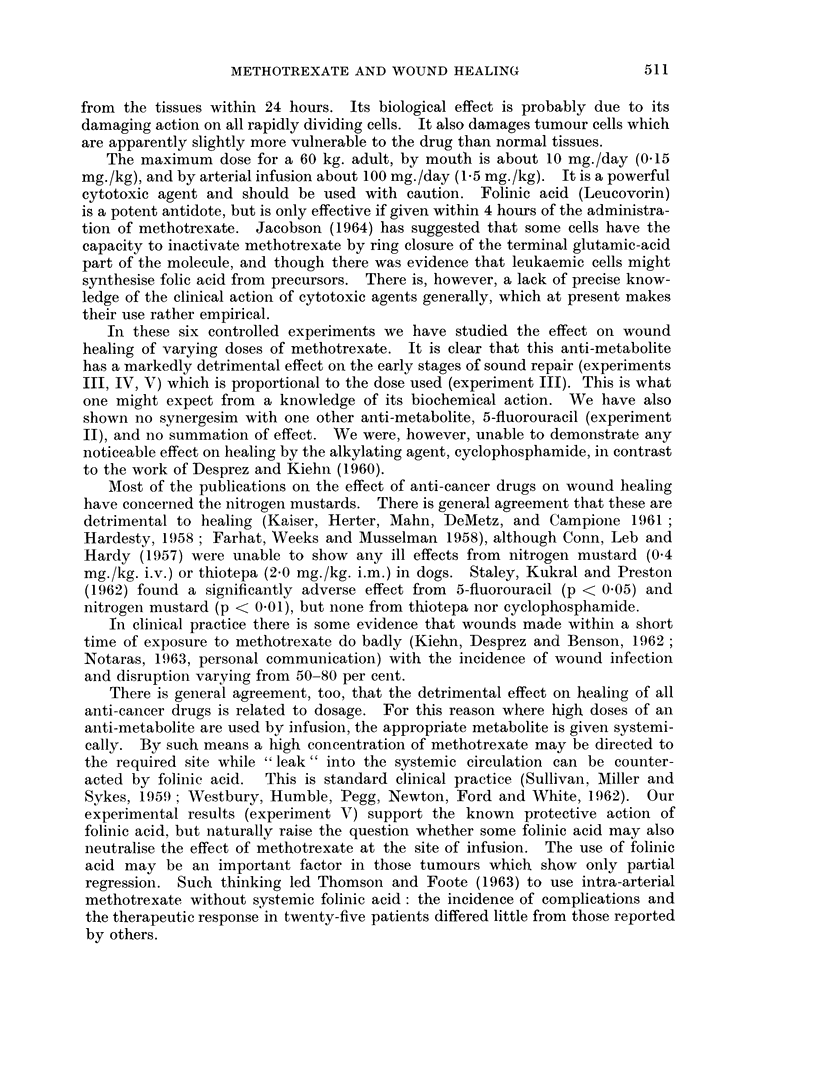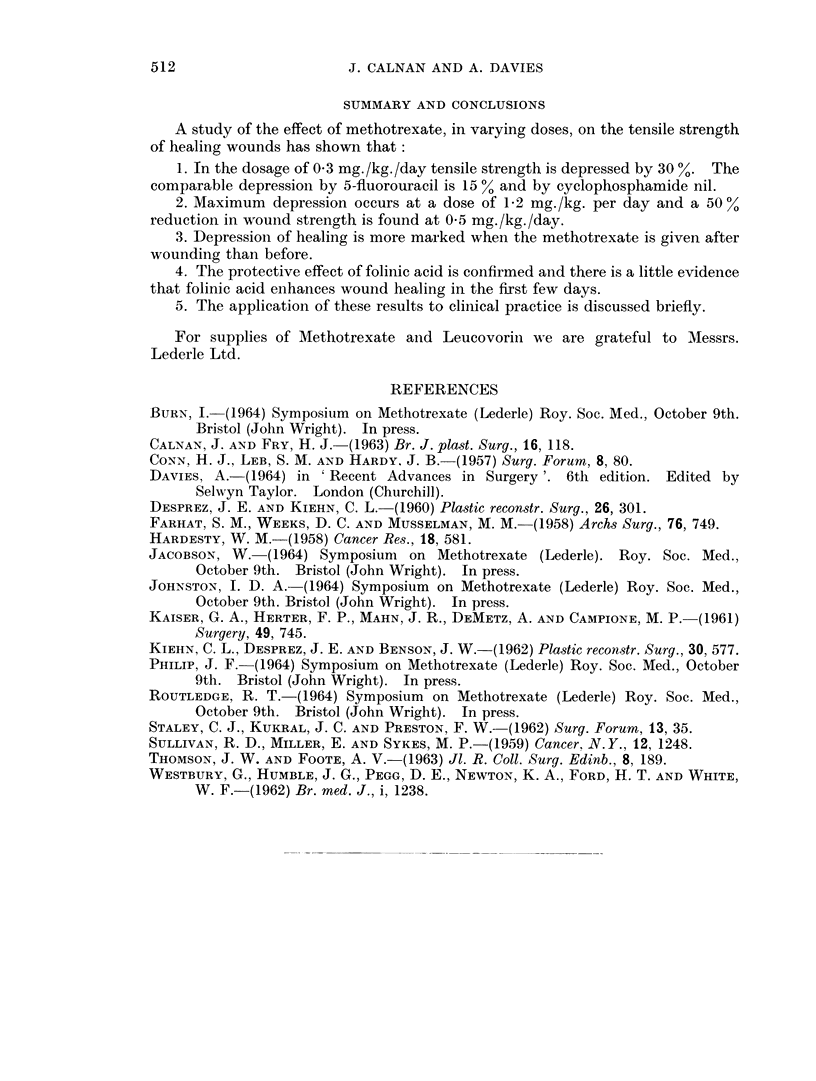# The effect of methotrexate (amethopterin) on wound healing: an experimental study.

**DOI:** 10.1038/bjc.1965.61

**Published:** 1965-09

**Authors:** J. Calnan, A. Davies


### THE EFFECT OF METHOTREXATE (AMETHOPTERIN) ON WOUND HEALING: AN EXPERIMENTAL STUDY

J. CALNAN AND A. DAVIES*

*From the Department of Surgery, Postgraduate Medical School of London (University of London) London, W.12.*

Received for publication April 30, 1965

THE value of cytotoxic agents in the treatment of cancer is now established, and the comparative place of the many available agents in different tumours is now becoming clear (Davies 1964). In this respect, methotrexate is accepted as the drug of choice for epithelioma of the head and neck, although it is less clear how and when it should be employed.

Given intra-arterially and used as the sole agent in regional infusion therapy, about 10 % of tumours are cured and nearly 50 % show partial regression (Burn, 1964; Johnston, 1964). Although these results are full of promise they are nonetheless disappointing. Some form of adjuvant therapy has therefore been recommended. Philip (1964) has shown that the cure rate can be improved when methotrexate is combined with radiotherapy, and Routledge (1964) has used it before surgical excision of a tumour. Although improved survival figures can be shown by combined techniques, they are small in relation to the numbers who show local recurrence of their tumours. A new method of treatment, in which methotrexate was infused intra-arterially before and after surgical excision of the tumour, seemed worthy of trial. Before starting such a trial we wished to study the effect of methotrexate on wound healing experimentally.

### MATERIALS

We chose white Norwegian (" Wistar ") rats because they had been used in the study of wound healing many times before, and the findings appear to be comparable with those in man. All were young (4–6 weeks) females.

### METHODS

Four standard wounds were made on the backs of a group of rats of similar weight and the animals allocated at random to one form of treatment. Adequate controls were used in every experiment. The tensile strength of the wounds was measured 3, 5 and 7 days after wounding, using a Sandblom tensiometer (Calnan and Fry, 1963). The measurements were made without reference to treatment, and the data examined by an analysis of variance. The methotrexate, or other drug, was freshly made up and given intraperitoneally in $\frac{1}{2}$ c.c. volumes each day. The rats were housed in separate cages at room temperature and fed on a pellet diet (Dixon's No. 7) with water *ad libitum.*

Six experiments were performed.

* Present address: St. Mark's Hospital, City Road, London, E.C.1.

### EXPERIMENTS AND RESULTS

### *I. Effect of various cytotoxic agents on wound healing*

Number of rats = 24, mean weight 154 g. (S.D. 8·5)
Experimental design = incomplete random block.

Treatments : 1. Methotrexate : 0·375 mg./kg./day
2. 5-Fluorouracil : 7·5 mg./kg./day
3. Cyclophosphamide : 7·5 mg./kg./day
4. Control : Saline only.

The tensile strength of the standard wounds, measured at 5 days, are shown in Fig. 1. It is clear from this that methotrexate depresses wound healing to a

FIG. 1.—Chart of mean tensile strength of wounds in rats at 5 days. The effect of methotrexate is significant ($P < 0\cdot05$) (experiment I).

significant degree ($P < 0\cdot5$), and cyclophosphamide not at all. When one considers the loss of weight of the rats during the experiment, a reverse order is found (Table I). Some loss of weight (due to wounding, daily injections, and a

TABLE I.—*Weight Changes of Wounded Rats*

| Treatment | Mean weights of rats at start of experiment (g.) | Mean *loss* of weight after 5 days treatment (g.) |
|---|---|---|
| 5-Fluorouracil | 154·2 | 22·0 |
| Cyclophosphamide | 155·3 | 29·5 |
| Methotrexate | 157·3 | 16·8 |
| Control | 150·6 | 13·8 |

body dressing) is common, but the loss when cyclophosphamide is given is more than twice that of the controls.

*II. The inter-actions between cytotoxic drugs*

Number of rats = 56, mean weight 168 g. (S.D. 10·4)
Experimental design = Factorial

Treatments : 1. Methotrexate : 0·125 mg./kg./day
2. 5-Fluorouracil : 2·5 mg./kg./day
3. Cyclophosphamide : 2·5 mg./kg./day
4. Saline only

Tensile strength measured at 5 days after wounding.

The results in Table II demonstrate that, although the tensile strengths of the treated wounds are less than those of the controls, there is little difference between various treatments. Methotrexate appears to have a more depressing effect on wound healing than the other drugs but the difference is not statistically significant. In general the effect on wound healing agrees with the findings of experiment I and does not indicate any interaction between the three cytotoxic agents tested.

TABLE II.—*Tensile Strength of 5-Day Wounds*

(Factorial Experiment)

| Treatment (for dosage see text) | Tensile strength in grams force (S.E. 18·7) Methotrexate Given | Not given |
|---|---|---|
| 5-Fluorouracil plus cyclophosphamide | 107·6 | 135·6 |
| 5-Fluorouracil | 110·0 | 126·7 |
| Cyclophosphamide | 122·0 | 148·7 |
| Methotrexate only | 105·6 | — |
| No drug : controls | — | 154·7 |

III. *Dose-response curve of methotrexate*

Number of rats = 20, mean weight 161 g. (S.D. 9·9).
Experimental design = incomplete random block

Treatments (4) : Methotrexate 0·125 mg./kg./day
0·25 mg./kg./day
0·5 mg./kg./day
1·0 mg./kg./day

Tensile strength measured at 5 days.

The effect of increasing doses of methotrexate on wound tensile strength is shown in Fig.2. Wound healing appears to be depressed significantly at a dose of 0·3 mg./kg., and markedly affected at 0·9 mg./kg.—corresponding to the oral systemic and intra-arterial infusion doses used in man.

IV. *Effect of pre- and post-operative methotrexate on wound healing*

Number of rats = 24, mean weight 144 g. (S.D. 9·3).
Experimental design = incomplete random block.

Treatments : Methotrexate 0·05 mg./kg./day for 5 days before wounding : nil after.
Methotrexate 0·05 mg./kg./day for 5 days before and 5 days after wounding.
Methotrexate 0·05 mg./kg./day for 5 days after wounding only.
Controls : no drug : saline only

FIG. 2.—Dose-response curve with methotrexate on the tensile strength of 5-day-old wounds in rats (experiment III).

The results in Fig. 3. show that the post-operative exhibition of methotrexate, in this rather small dosage, has a more depressing effect on healing than when used before operation.

V. *The protective effect of folinic acid*

Number of rats = 20, mean weight 192 g. (S.D. 15).
Experimental design = incomplete random block.

Treatments : Methotrexate 0·5 mg./kg./day
{Methotrexate 0·5 mg./kg./day
{Folinic acid (Leucovorin) 2·5 mg./kg./day
Folinic acid (Leucovorin) 2·5 mg./kg./day
Controls : Saline only.

The results are shown in Fig. 4. It is clear that methotrexate in this large dosage depressed wound healing when the tensile strength was measured at 3 and 7 days, but that Leucovorin (folinic acid) will protect completely. It is interesting

FIG. 3.—The effect of pre- and post-operative methotrexate (0·05 mg./kg.) on the tensile strength of 5-day-old wounds in rats. The post-operative use of methotrexate causes a significant depression of tensile strength ($P < 0\cdot05$).

to note that Leucovorin when given alone appeared to enhance wound healing in the early stages to a significant degree ($P < \cdot01$) but this effect is short-lived.

VI. *The effect of Leucovorin (folinic acid) on wound healing*

Number of rats = 16, mean weight 73 g. (S.D. 5).
Experimental design = incomplete random block.

Treatments : Leucovorin 7·5 mg./kg./day subcutaneously in ½ ml.
0·75 mg./kg./day
0·075 mg./kg./day
Controls : saline injections.

The wounds were measured at 5 days but no statistically significant differences were noted. The mean tensile strength in each group, however, was related to the dose of folinic acid—the larger the dose, the greater the tensile strength (Table III).

TABLE III.—*Effect of Folinic Acid (Leucovorin) on 5-Day Wounds*

| Folinic acid | Mean tensile strength (g. force) & S.E. |
|---|---|
| 15 mg./kg. . | 250 ± 12·8 |
| 1·5 mg./kg. . | 235 ,, ,, |
| 0·15 mg./kg. . | 190 ,, ,, |
| Controls . . | 227 ,, ,, |

The effect at 5 days is not statistically significant (compare with Fig. 4).

FIG. 4.—The protective effect of folinic acid (Leucovorin). At a dosage of 0·5 mg./kg. methotrexate causes a significant depression of wound healing ($P < 0·01$) but folinic acid (2·5 mg./kg.) will prevent this.

### DISCUSSION

Methotrexate (Amethopterin) is a potent folic acid antagonist and cytotoxic agent, with the formula 4-amino-$N^{10}$-methyl-pteroyl-glutamic acid. Its action is to prevent the reduction of folic acid to tetrahydrofolic acid by uniting with dihydrofolic reductase, the substance which catalyses the process. The affinity of methotrexate for the enzyme is about 100,000 times greater than that of the normal substrate.

Folic acid is an essential starting substance for the formation of analogues whose function is to introduce single carbon atoms into a number of molecules. It is through this mechanism that folic acid is involved in nucleic acid and purine synthesis. Methotrexate blocks this process, and probably produces most of its effects by interfering with purine synthesis.

When given intravenously methotrexate is excreted in the urine and cleared from the tissues within 24 hours. Its biological effect is probably due to its damaging action on all rapidly dividing cells. It also damages tumour cells which are apparently slightly more vulnerable to the drug than normal tissues.

The maximum dose for a 60 kg. adult, by mouth is about 10 mg./day (0·15 mg./kg), and by arterial infusion about 100 mg./day (1·5 mg./kg). It is a powerful cytotoxic agent and should be used with caution. Folinic acid (Leucovorin) is a potent antidote, but is only effective if given within 4 hours of the administration of methotrexate. Jacobson (1964) has suggested that some cells have the capacity to inactivate methotrexate by ring closure of the terminal glutamic-acid part of the molecule, and though there was evidence that leukaemic cells might synthesise folic acid from precursors. There is, however, a lack of precise knowledge of the clinical action of cytotoxic agents generally, which at present makes their use rather empirical.

In these six controlled experiments we have studied the effect on wound healing of varying doses of methotrexate. It is clear that this anti-metabolite has a markedly detrimental effect on the early stages of sound repair (experiments III, IV, V) which is proportional to the dose used (experiment III). This is what one might expect from a knowledge of its biochemical action. We have also shown no synergesim with one other anti-metabolite, 5-fluorouracil (experiment II), and no summation of effect. We were, however, unable to demonstrate any noticeable effect on healing by the alkylating agent, cyclophosphamide, in contrast to the work of Desprez and Kiehn (1960).

Most of the publications on the effect of anti-cancer drugs on wound healing have concerned the nitrogen mustards. There is general agreement that these are detrimental to healing (Kaiser, Herter, Mahn, DeMetz, and Campione 1961 ; Hardesty, 1958 ; Farhat, Weeks and Musselman 1958), although Conn, Leb and Hardy (1957) were unable to show any ill effects from nitrogen mustard (0·4 mg./kg. i.v.) or thiotepa (2·0 mg./kg. i.m.) in dogs. Staley, Kukral and Preston (1962) found a significantly adverse effect from 5-fluorouracil ($p < 0·05$) and nitrogen mustard ($p < 0·01$), but none from thiotepa nor cyclophosphamide.

In clinical practice there is some evidence that wounds made within a short time of exposure to methotrexate do badly (Kiehn, Desprez and Benson, 1962 ; Notaras, 1963, personal communication) with the incidence of wound infection and disruption varying from 50–80 per cent.

There is general agreement, too, that the detrimental effect on healing of all anti-cancer drugs is related to dosage. For this reason where high doses of an anti-metabolite are used by infusion, the appropriate metabolite is given systemically. By such means a high concentration of methotrexate may be directed to the required site while "leak" into the systemic circulation can be counteracted by folinic acid. This is standard clinical practice (Sullivan, Miller and Sykes, 1959 ; Westbury, Humble, Pegg, Newton, Ford and White, 1962). Our experimental results (experiment V) support the known protective action of folinic acid, but naturally raise the question whether some folinic acid may also neutralise the effect of methotrexate at the site of infusion. The use of folinic acid may be an important factor in those tumours which show only partial regression. Such thinking led Thomson and Foote (1963) to use intra-arterial methotrexate without systemic folinic acid : the incidence of complications and the therapeutic response in twenty-five patients differed little from those reported by others.

SUMMARY AND CONCLUSIONS

A study of the effect of methotrexate, in varying doses, on the tensile strength of healing wounds has shown that :

1. In the dosage of 0·3 mg./kg./day tensile strength is depressed by 30 %. The comparable depression by 5-fluorouracil is 15 % and by cyclophosphamide nil.
2. Maximum depression occurs at a dose of 1·2 mg./kg. per day and a 50 % reduction in wound strength is found at 0·5 mg./kg./day.
3. Depression of healing is more marked when the methotrexate is given after wounding than before.
4. The protective effect of folinic acid is confirmed and there is a little evidence that folinic acid enhances wound healing in the first few days.
5. The application of these results to clinical practice is discussed briefly.

For supplies of Methotrexate and Leucovorin we are grateful to Messrs. Lederle Ltd.

REFERENCES

Burn, I.—(1964) Symposium on Methotrexate (Lederle) Roy. Soc. Med., October 9th. Bristol (John Wright). In press.
Calnan, J. and Fry, H. J.—(1963) *Br. J. plast. Surg.*, **16**, 118.
Conn, H. J., Leb, S. M. and Hardy. J. B.—(1957) *Surg. Forum*, **8**, 80.
Davies, A.—(1964) in 'Recent Advances in Surgery'. 6th edition. Edited by Selwyn Taylor. London (Churchill).
Desprez, J. E. and Kiehn, C. L.—(1960) *Plastic reconstr. Surg.*, **26**, 301.
Farhat, S. M., Weeks, D. C. and Musselman, M. M.—(1958) *Archs Surg.*, **76**, 749.
Hardesty, W. M.—(1958) *Cancer Res.*, **18**, 581.
Jacobson, W.—(1964) Symposium on Methotrexate (Lederle). Roy. Soc. Med., October 9th. Bristol (John Wright). In press.
Johnston, I. D. A.—(1964) Symposium on Methotrexate (Lederle) Roy. Soc. Med., October 9th. Bristol (John Wright). In press.
Kaiser, G. A., Herter, F. P., Mahn, J. R., DeMetz, A. and Campione, M. P.—(1961) *Surgery*, **49**, 745.
Kiehn, C. L., Desprez, J. E. and Benson, J. W.—(1962) *Plastic reconstr. Surg.*, **30**, 577.
Philip, J. F.—(1964) Symposium on Methotrexate (Lederle) Roy. Soc. Med., October 9th. Bristol (John Wright). In press.
Routledge, R. T.—(1964) Symposium on Methotrexate (Lederle) Roy. Soc. Med., October 9th. Bristol (John Wright). In press.
Staley, C. J., Kukral, J. C. and Preston, F. W.—(1962) *Surg. Forum*, **13**, 35.
Sullivan, R. D., Miller, E. and Sykes, M. P.—(1959) *Cancer, N.Y.*, **12**, 1248.
Thomson, J. W. and Foote, A. V.—(1963) *Jl. R. Coll. Surg. Edinb.*, **8**, 189.
Westbury, G., Humble, J. G., Pegg, D. E., Newton, K. A., Ford, H. T. and White, W. F.—(1962) *Br. med. J.*, i, 1238.